# Pruritus in Autoimmune and Inflammatory Dermatoses

**DOI:** 10.3389/fimmu.2019.01303

**Published:** 2019-06-21

**Authors:** Claudia Zeidler, Manuel Pedro Pereira, Flavien Huet, Laurent Misery, Kerstin Steinbrink, Sonja Ständer

**Affiliations:** ^1^Department of Dermatology and Center for Chronic Pruritus, University Hospital Münster, Münster, Germany; ^2^Department of Dermatology, University Hospital of Brest, Brest, France; ^3^Univ. Brest, LIEN, Brest, France

**Keywords:** itch, pruritus, psoriasis, atopic dermatitis, IL31, substance P, bullous pemphigoid

## Abstract

Pruritus in autoimmune and inflammatory dermatoses is a common symptom that can be severe and affect the quality of life of patients. In some diseases, pruritus is related to disorders activity and severity or may occur independent of the disease. Despite the high prevalence, the symptom is still underrated and there are only a few trials investigating the efficacy of drugs for disease-specific pruritus. In this review, the characteristics and possible pathomechanisms of pruritus in various dermatoses like autoimmune bullous diseases, connective tissue diseases as well as autoimmune-associated dermatoses (atopic dermatitis, psoriasis vulgaris) is illustrated. Additionally, studies analyzing the antipruritic treatment are discussed. Summarizing, the prevalence of pruritus in these diseases demonstrates the importance for symptom recognition and the need for an efficient antipruritic therapy.

## Introduction

Autoimmune diseases occur due to a deregulated immune response directed to the body own tissues. Cutaneous manifestations may occur secondarily to systemic autoimmune conditions such as lupus erythematosus (LE) or systemic sclerosis (SSc) or result from primary dermatoses as e.g., in autoimmune bullous diseases (AIBD) or in vitiligo (1). A feature of autoimmune disorders is the occurrence of an exacerbated inflammation owing to an enhanced release of pro-inflammatory mediators. One common symptom is pruritus which is listed by the Global Burden of Disease project as one of the 50 most common interdisciplinary symptoms leading to high burden levels (2). Chronic pruritus (CP) is often accompanied by paresthetic sensations like warmth, burning and stinging (3). Physicians should be alert to this debilitating symptom and offer patients with adequate antipruritic care. In this review we provide an overview of the most relevant autoimmune dermatoses focusing on pruritus, including, pathophysiological mechanisms, clinical features and when available anti-pruritic treatment options (Table 1). Atopic dermatitis (AD) and psoriasis vulgaris are included since autoimmunity phenomena have been observed in these (4–7).

**Table 1:** Overview of diseases and pruritus-specific data.

**Disease**	**Clinical manifestations in the skin**	**Characterics of pruritus**	**Expected pathomechanism of pruritus**	**Studies for treatment of disease related pruritus**
AD	Highly variable: pruritic, red, scaly, and crusted lesions, lichenified plaques, Xerosis cutis	Pr: almost 100%, severe pruritus	High IL31 Levels, bradykinin, TSLP, SP, CGRP, NGF	UVB, CyA, Dupilumab, Nemolizumab, Tezepelumab, Crisaborole, apremilast
Pso	Well-demarcated, erythematous plaques with thick silvery scale	Pr: 60–90%	Increased intraepidermal nerve fiber density, overexpression of SP, TRPV1, IL31, TRP melastatin 8, TRP vanilloid 3	Anti-IL17, JAK inhibitors, adalimumab, apremilast, ustekinumab, anti-IL23
BP	Tense blisters on an erythematous base or normal skin, but also non-bullous variant with pruritic, erythematous or urticarial lesions	Typical symptom	Elevated IL31, hyperactive basophils	N/A
PV	Flaccid, fragile blisters, rythematous, oozing and subsequently crusted erosions, erosions of mucosa	Rare	Inflammatory infiltrate with eosinophils	N/A
DH	Grouped herpetiform papulovesicles with erosions and crusts, with symmetric distribution predominantly in the shoulder and gluteal region as well as on the elbows and knees	Often, sleep disturbance	Hyperactive mast cells, higher expression of IL31 receptors	N/A
SSc	Skin fibrosis, Raynaud's phenomenon, telangiectasia, ulcers, calcinosis	Pr: 40–65%,pain, burning, stingling	Neuropathic component	N/A
Mor	Initial inflammatory, erythematous patch followed by sclerotic changes and subsequent atrophy	Pruritus is activity marker	Inflammation, neuropathic component	N/A
CLE	Diverse skin involvement depending on subtype	Pr: 75%	Possibly inflammation	N/A
DM	Gottrons papules (erythematous to violaceous papules over MCP joints), heliotrope eruption (violaceous eruption in the upper eyelids) and facial erythema	Median pruritus VAS: 3.80	Decreased density of epidermal nerves and formed complex tufts	Apremilast
SS	Xerosis	Pr: 42–53%	Xerosis	N/A
Vit	Depigmented areas	Pr: 20%	Elevated histamine, neurogenic mechanism with release of melanocyte- toxic neuropeptides of cutaneous peripheral nerve endings	N/A

*AD, atopic dermatitis; Pso, Psoriasis vulgaris; BP, bullous pemphigoid; PV, pemphigus vulgaris; DH, Dermatitis herpetiformis; SSc, systemic sclerosis; Mor, Morphea; CLE, Cutaneous Lupus erythematosus; DM, Dermatomyositis; SS, Sjögrens Syndrome; Vit, Vitiligo; Pr, prevalence; IL, interleukine; TSLP, thymic stromal lymphopoietin; SP, substance P; CGRP, calcitonin gene-related peptide; NGF, nerve growth factor; TRPV1, transient receptor potential vanilloid 1; TRP, transient receptor potential; CyA, ciclosporine A; JAK, janus kinase; N/A, not available*.

## Atopic Dermatitis

AD is a common disease which affects about 20% of children and 5% of adults (8). The pathophysiological mechanisms are an immune deviation toward T helper cells 2 cells secreting predominantly IL4, IL5, and IL13 in in the initiation phase with consequent increased IgE production, a deficient skin barrier function, an abnormal microbial colonization and a neurogenic inflammation (9). Severe pruritus appears in almost all patients (10), correlating with severity of AD (11). Ninety-three percent of patients reported to scratch often or very often, and perceive scratching as pleasurable which lead to itch-scratch cycle (12). Further, stinging and burning were described, suggesting a neuropathic component. Pruritus impacts strongly the sleep and the quality of live (QoL), which leads to a higher rate of anxiety, depression and suicidal ideation (13). Among pruritus mediators, the role of histamine is restrained, as indicated by the inefficacy of anti-histamine treatment (14). Proteinase activated receptor-2 expressed by keratinocytes and cutaneous free nerve endings is involved in the pruritus pathway of AD (15). Recently, ORAI1, a channel mediating store-operated Ca^2^+ influx which is required for NFAT-dependent cytokines expression, was shown to be an regulator of AD cytokine and itch-causing compound (16). Activated nerves fibers release neuropeptides like CGRP and substance P (SP). IL31 produced by Th2 cells is overexpressed in lesional skin and in serum (17) and correlates with the disease severity (18). Thus, IL31 induces directly itching via its receptor which is expressed on keratinocytes and nerve fibers (NF) (19). Also, environment plays a role. Various organic components of pollutant activate the aryl hydrocarbon receptor in keratinocytes and stimulate artemin, a neurotrophic factor which is responsible for epidermal hyperinnervation (20). This may be partly responsible for itch sensitization, although a new study questions this affirmation (21, 22).

A few studies have investigated the treatment of AD-associated pruritus. In addition to known therapies like narrow band UVB, which lead to a decreased pruritus intensity in 90% of patients with AD (23), or cyclosporine which has shown a significant reduction of pruritus intensity and decreased IL31 serum levels (24) also new treatment regimens have been investigated for their antipruritic effect in AD. Dupilumab, a monoclonal antibody (mAb) binds to the alpha subunit of the IL4 receptor, thereby inhibiting the binding of IL4 and IL13. In placebo controlled studies, dupilumab significantly reduced pruritus (44–51% reduction) (25). Tralokinumab and lebrikizumab target anti-IL13 leading to AD improvement and decrease of pruritus. Nemolizumab, a IL-31 receptor mAb, inhibits the IL31 signaling and reduced the intensity of pruritus in a phase II study [up to 90% in a 52 week (26)]. Tezepelumab is a mA targeting TSLP. In light of the role of TSLP in atopic pruritus, tezepelumab seems very interesting. Nevertheless, in a phase II study, tezepelumab showed limited efficacy on pruritus and inflammatory signs (27).

Phosphodiesterase 4 (PDE4) is an enzyme that regulates cAMP levels and thereby pro-inflammatory cytokines involved in AD. Crisaborole, a new topical PDE4 inhibitorapproved in AD, showed improvement in pruritus (28). In a phase II study, apremilast, an oral PDE4 inhibitor, showed modest efficacy in AD and a decrease in pruritus but no significant compared to placebo (25).

JAK/STAT pathways are activated by typical AD cytokines such as IL,4, IL13, or IL31. Inhibitors are being evaluated for oral treatment of AD. First results seem promising (29).

The level of SP is involved in neurogenic inflammation and increased pruritus. Though effective in prurigo nodularis, in phase II study with oral NK1-receptor antagonist, serlopitant did not show improvement in pruritus of patients with AD.

## Psoriasis Vulgaris

Psoriasis was considered as a non-pruritic dermatosis. Nowadays, numerous studies have clearly documented that pruritus is a very frequent symptom of psoriasis (30) with a prevalence of 60–90% in patients suffering from psoriasis and a mean severity around 6/10 points on visual analog scale (VAS) (30). Pruritus is higher in patients with dry skin, stress (31) or the presence of depression and anxiety disorders (32). CPcorrelates also with the use of antacids, angiotensin receptor blockers, angiotensin enzyme converting inhibitors and beta-blockers (33).

A large majority of patients with psoriasis consider pruritus as the most bothersome symptom of their disease (34). Patients with pruritus report a greater reduction in their health-related QoL, including the ability to sleep, compared to those without pruritus. The severity of pruritus correlates with the degree of QoL impairment. The pathogenesis of pruritus in psoriasis is poorly known since the main cytokines involved in psoriasis are not known to be pruritogenic. The major concept of pruritus origin is focused on neurogenic inflammation, through the release of neuropeptides from nerve endings, in association with a modified innervation density in psoriasis as well as an abnormal functioning of the peripheral opioid system (22). Overexpression of genes, such as phospholipase A2 IVD, SP, voltage-gated sodium channel 1.7, transient receptor potential (TRP) vanilloid 1, IL17A, IL23A, IL31, TRP melastatin 8, TRP vanilloid 3, phospholipase C, and IL36α/γ, has been shown in pruritic psoriatic skin (35). The efficacy of treatments of psoriasis is commonly judged on their effects on visible skin lesions but it might be more important to consider the resolution of pruritus, since it is the first preoccupation of the patients (34) and because there is no specific antipruritic therapy to treat pruritus in psoriasis. A systematic meta-analysis has evaluated the effect of systemic psoriasis treatments on psoriatic pruritus (36). Anti-IL17 showed the greatest antipruritic effect. JAK inhibitors were more effective than adalimumab, which was more effective than apremilast. Other studies could not be included in the meta-analysis for methodological reasons. Nevertheless, UVB phototherapy and cyclosporine were also noted to be effective. Some studies showed the favorable effects of ustekinumab (anti-IL12) (37) and anti-IL23 (38) on pruritus. A topical inhibitor of nerve growth factor (NGF) receptor has also been shown to be effective for the treatment of pruritus due to psoriasis (39).

## Autoimmune Bullous Diseases

AIBD are a heterogeneous group of severe dermatoses characterized by the presence of autoantibodies against cutaneous adhesion molecules (40).

## Bullous Pemphigoid

Pruritus in the elderly patients is a frequent complaint and bullous pemphigoid (BP) is a rare but an important differential diagnosis. Recent data suggest that pruritus of BP might be linked to elevated levels of cutaneous IL31 (41). Histologically, BP is characterized by a dense infiltrate of eosinophils which produce and release IL31 (42). IL31 is well-known for the induction of pruritus via inflammatory and neuronal mechanisms (43). In addition, histamine released from basophils seems to play a role. Basophils might be present in BP lesions, too (44). Compared to healthy control basophils, circulating peripheral basophils from BP patients degranulate and release histamine when incubated with BP180 (45).

In BP, all clinical phenotypes are associated with severe pruritus (46). Pruritus can precede the development of the lesions (47). In a preclinical stage, pruritus without visible skin lesions can be the only manifestation of BP (48, 49). Interestingly, there are also many reports of atypical, mainly non-bullous, clinical variants of BP associated with IgG autoantibodies against BP180 and BP230 associated with pruritus as the common leading clinical symptom (50). These subtypes are heterogeneous and include eczematous, erythematous plaques, urticarial, papular and/or nodular skin lesions (50). However, a small study including patients with chronic pruritic skin disorders (*n* = 78) and patients with non-inflammatory skin disease (*n* = 93) failed to detect specific autoantibodies (40). Elderly patients with pruritus may present with a broad range of underlying diseases including metabolic diseases, drug intake and neuropathic conditions (51). To address the specific question on the prevalence of atypical BP as an origin of CP in the elderly, a large population of patients' needs to be investigated.

**Figure 1:**
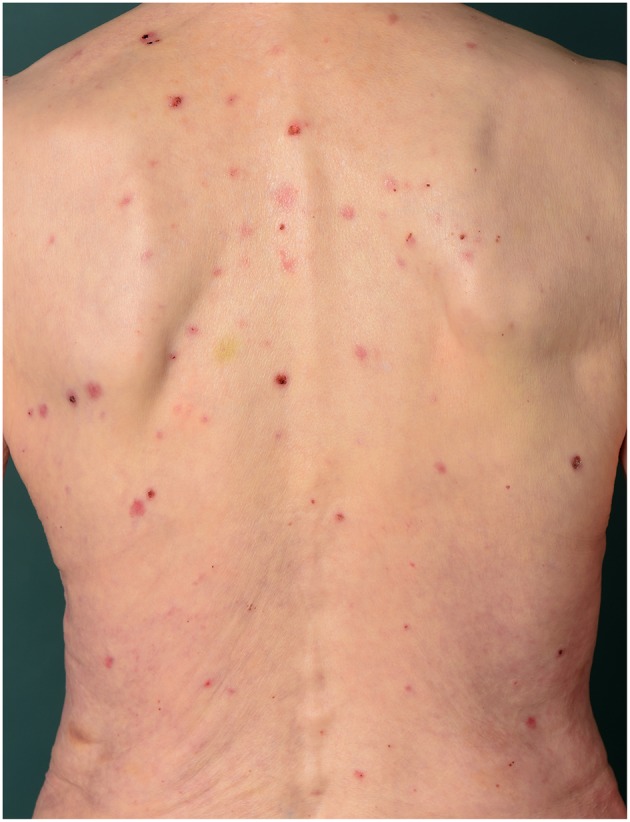
Seventy-eight-year old female patient with BP. Excoriations, bleeding and crusts caused by scratching can be observed.

Scratching typically accompanies pruritus in BP. Subsequently, patients develop excoriations, bleeding and crusts (Figure 1). Some can even develop chronic prurigo lesions due to prolonged scratching behavior (52). Patients experience pruritus to all day and night times without a preference and aggravation after emotional stress (46).

The current therapy recommendations do not outline specific antipruritic therapies besides the immunosuppressive therapies (52). Pruritus parallels the disease course in BP. Accordingly, cessation of pruritus is one criterion of disease control in BP (49) and monitoring of pruritus is an important step which can be done using the Subjective Bullous Pemphigoid Disease Area Index pruritus score (49). For patients with impaired mental functioning, indirect assessment of pruritus via presence of signs of scratching and sleep disturbance is suggested (49).

## Pemphigus Group

Pemphigus is a potentially life-threatening AIBD and characterized by flaccid fragile blisters and erosions of the skin and/or mucous membranes. In contrast to BP, pruritus is less frequently present and with lower intensity in the pemphigus group (46). The most common subjective symptoms reported by patients with pemphigus vulgaris are burning (83.1%), pain (68.4%), and pruritus (47.5%) (53). Histopathologically, a suprabasal, akantholytic separation and blistering with a retention of basal keratinocytes along the basement membrane zone, and sparse inflammatory infiltrate in the dermis with eosinophils can be observed in pemphigus. The inflammation might be of great relevance for the induction of pruritus. Pemphigus foliaceus is another disease of this group. Here, pruritus occurs in more than half of the patients (61%) (54). The histopathological characteristic findings include intraepithelial cleavage with acantholysis beneath the stratum corneum and a dermal inflammation, predominantly with neutrophils, mast cells and plasma cells (54). Although there is little systematic data on pruritus in the pemphigus group, the parameter pruritus contributes to the assessment whether the disease is controlled or not (55).

## Dermatitis Herpetiformis (Duhring's Disease)

Dermatitis herpetiformis (DH) is found more often in young adults and children and often associated with coeliac disease. It is characterized by granular deposits of IgA in dermal papillae, as well as deposits of other immunoglobulins and complement components (56). Pruritus is common and often the first symptom. The intensity of pruritus is high with a mean intensity of pruritus of 8/10 on a numerical rating scale. 2/3 of patients have sleep disorders related to pruritus (57). In the same study group the serum IL31 levels were reduced in DH compared to a healthy control group. This was surprising, because IL31 levels are increased in other pruritic dermatoses like AD (58) and psoriasis vulgaris (59). One explanation could be that mast cells are hyperactive which leading to a higher expression of IL31 receptors, which may be the reason for the low serum concentration of IL31 (57). Usually, pruritus reliefs during treatment but further studies on antipruritic effects are missing.

## Connective Tissue Diseases

### Systemic Sclerosis

The manifestations of SSc are diverse. Abnormalities of the circulation (most notably Raynauds phenomenon) and involvement of multiple organ systems, including the renal, pulmonary, cardiac, and gastrointestinal systems due to fibrosis and vasculopathy development, are most prominent. Skin involvement is characterized by variable extent and severity of skin thickening and hardening with edematous swelling and erythema. With a prevalence of 40–65%, pruritus is a common symptom of SSc, which occurs not only in the affected areas but also often on the extremities or generalized (60). In addition to pruritus, patients experience stinging, burning and pain, which suggests that pruritus in SSc has a neuropathic component (61) caused by compression of small NF by thickened collagen. There are no data which investigate the antipruritic effect by an effective therapy of SSc. However, it might be assumed that modified NF necessitates a specific antipruritic therapy.

### Morphea

Morphea is an idiopathic, inflammatory disorder. The initial sign is often an inflammatory, erythematous patch followed by sclerotic dermal changes and subsequent atrophy. There are a lot of variants describing the clinically based division into circumscribed (65%), generalized (8%), linear (6%), and mixed forms (62). Pruritus is a distressing symptom in morphea, which causes a reduction in QoL (63). Interestingly, pruritus leads to a greater restriction than the location of lesions in cosmetically or functionally sensitive sites (63). In particular, in an active morphea, pruritus is noticeable, so that it is proposed as an activity marker. The cause of pruritus has not been elucidated. Based on the previously described observation, it can be concluded that inflammation with an infiltrate from lymphocytes, plasma cells, eosinophils, and mast cells is an important factor in the development of pruritus. However, a neuropathic component in the later stage of the morphea by compression of small NF is also conceivable. Further investigations on antipruritic therapy are not available.

### Lupus Erythematosus/Dermatomyositis/Sjögren Syndrome

#### Cutaneous Lupus Erythematosus

Cutaneous LE (CLE) shows diverse skin manifestations depending on the present subtype. The classification is based on clinical characteristics like photosensitive lesions which lead to hyperpigmentation, scarring and hair loss in discoid lupus erythematosus or purple plaques/nodules and edematous skin mainly in the acral regions in chilblain lupus {Patel 2013 #13}. The prevalence of pruritus is 75% in patients suffering from CLE (64). The severity was widely ranged from mild (62.1%), moderate (23.1%), and severe (14.8%) (64). The intensity of pruritus correlates with the activity of skin lesions suggesting that the pathomechanism of pruritus is related to inflammation. However, there are no pruritus specific studies regarding the pathophysiology. Immunosuppressive therapy like use corticosteroids and steroid-sparing agents can relieve pruritus (65, 66). However, pruritus is a common side effect antimalarial drugs which are often used in the treatment of CLE (67).

### Sjögren Syndrome

Sjögren syndrome (SS) is a rare autoimmune disease characterized by chronic dryness of the mucous membranes (sicca's syndrome) and by chronic, progressive inflammation and exocrine gland insufficiency (68). The syndrome can be divided into a primary (cause unknown) and a secondary SS (association with connective tissue disease) (68). The prevalence of pruritus is 42–53% in both types of SS together. Patients suffering from SS and pruritus have a greater impairment of QoL and a higher rate of sleep disturbance (69). An important factor in the pathophysiology of pruritus in SS is xerosis cutis which is underlined by the fact that the most pruritic locations in SS are the shins—a very common location of xerosis induced pruritus (70).

### Dermatomyositis

Dermatomyositis (DM) is a rare systemic autoimmune disease characterized by immunological responses to vascular and muscle-derived proteins, resulting in inflammation of the skin and muscles, and typical skin lesions such as Gottron's papules (erythematous to violaceous papules over MCP joints), heliotrope eruption (violaceous eruption in the upper eyelids) and facial erythema (71). In addition, skin lesions may be accompanied by pruritus, which is more intense when compared to pruritus in CLE (median VAS DM: 3.80/10, median VAS CLE: 2/10). Additionally pruritus affects the QoL in patients with DM (72). Pathophysiologically, a small fiber neuropathy with a decreased density of epidermal nerves and formed complex tufts is thought to contribute to pruritus in DM (73). Recently, the antipruritic effect of apremilast in dermatomyositis induced scalp pruritus was reported. However, further studies on antipruritic effects of drugs are missing (65).

### Vitiligo

Vitiligo is a rare, congenital or acquired, localized skin pigmentation disorder. The disease is due to destruction and loss of melanocytes. There are a lot of hypotheses on the etiology of vitiligo including cytotoxic immune responses and presence of antibodies against melanocytes (74). However, vitiligo is not considered as a typical autoimmune disease by itself but patients have a genetic susceptibility to other autoimmune or autoinflammatory diseases like thyroid disease (75). Other hypotheses focus on a neurogenic mechanism with release of melanocyte- toxic neuropeptides of cutaneous peripheral nerve endings (Al-Abadie) which may also explain incidental occurrence of pruritus in vitiligo. Other authors speculate on histamine exhibiting a role in vitiligo associated pruritus (76). Elevated blood histamine levels have been found in patients with vitiligo and pruritus in comparison with matched controls (76). The prevalence of pruritus in vitiligo is 20% (77). Interestingly, active vitiligo was associated in 78.1% of patients with pruritus and Koebner phenomenon. Most patients reported a moderate pruritus (average 5/10 on NRS) and several sensory qualities of pruritus as tingling (82.7%), crawling (18.5%), and burning (18.5%). Concerning the QoL, those patients with pruritus and vitiligo had significant higher DLQI scores than those without it (78). The majority of patients with pruritus had daily activity (60.5%) and several had also sleep disturbances (39.5%). Improvement was reported by topical corticosteroids (55.6%) and oral antihistamines (9.9%).

### Management of Pruritus

**Figure 2:**
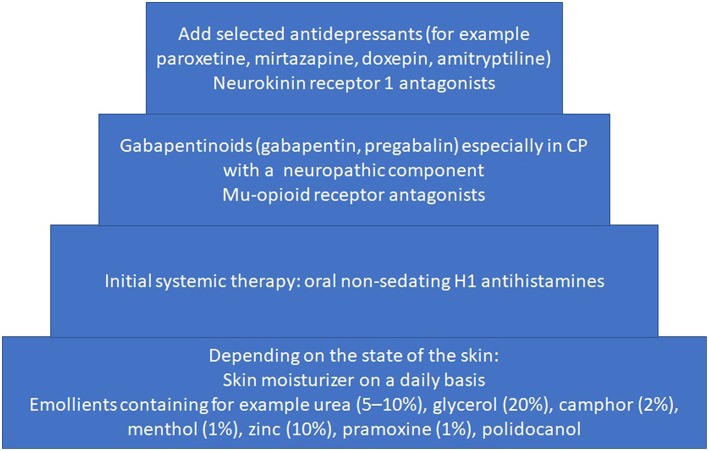
Stepwise therapeutic approach based on European S2k Guideline on Chronic Pruritus if causative treatment failed (79).

If there is no proven treatment and/or the causative therapy failed to relieve CP it is recommended to add stepwise an antipruritic treatment (Figure 2).

## Conclusion

Pruritus is a common symptom of autoimmune conditions affecting the skin. However, it is often overlooked in clinical routine and by the scientific community. Therefore, awareness on this issue should be raised. A better understanding of the pathophysiological mechanisms leading to pruritus and of the clinical features in these diseases is needed. Additionally, clinical trials analyzing the anti-pruritic efficacy of relevant substances for this patient group would be an important step in order to achieve a better care for these patients and ultimately improve their QoL.

## Author Contributions

CZ, MP, LM, FH, KS, and SS wrote the paper. CZ and MP prepared the figure. CZ prepared the table.

### Conflict of Interest Statement

LM: principal investigator for Abvie, Biogen, Janssen, Leo, Lilly, Pfizer, Sanofi; member of scientific advisory boards/consultant for Bayer, Celgene, Expanscience, Fresenius, Janssen, La Roche-Posay, Leo, Lilly, Menlo, Nestlé Skin Health, Novartis, Pierre Fabre, Sanofi; received grants from Beiersdorf, Bioderma, Celgene, Clarins, Expanscience, Johnson&Johnson, Nestlé Skin Health. FH: received finance of research project from Beiersdorf; consultant for Celgene; received material support from Novartis. KS: received research grants from Pfizer; principal investigator for Actelion; consultant for Leo. SS: principal investigator for Menlo Therapeutics Inc, Dermasence, Trevi Therapeutics, Galderma, Kiniksa, and Novartis; member of scientific advisory boards/consultant for Beiersdorf, Celgene, Galderma, Menlo Therapeutics Inc, NeRRe Therapeutics, Sienna, and Trevi Therapeutics, Novartis. The remaining authors declare that the research was conducted in the absence of any commercial or financial relationships that could be construed as a potential conflict of interest.
